# Using functional data analysis to understand daily activity levels and patterns in primary school-aged children: Cross-sectional analysis of a UK-wide study

**DOI:** 10.1371/journal.pone.0187677

**Published:** 2017-11-08

**Authors:** Francesco Sera, Lucy J. Griffiths, Carol Dezateux, Marco Geraci, Mario Cortina-Borja

**Affiliations:** 1 Department of Social and Environmental Health Research, London School of Hygiene and Tropical Medicine, London, United Kingdom; 2 Population, Policy and Practice Programme, UCL Great Ormond Street Institute of Child Health, London, United Kingdom; 3 Department of Epidemiology and Biostatistics, Arnold School of Public Health, University of South Carolina, Columbia, United States of America; University of Georgia, UNITED STATES

## Abstract

**Background:**

Temporal characterisation of physical activity in children is required for effective strategies to increase physical activity (PA). Evidence regarding determinants of physical activity in childhood and their time-dependent patterns remain inconclusive. We used functional data analysis (FDA) to model temporal profiles of daily activity, measured objectively using accelerometers, to identify diurnal and seasonal PA patterns in a nationally representative sample of primary school-aged UK children. We hypothesised that PA levels would be lower in girls than boys at play times and after school, higher in children participating in social forms of exercise (such as sport or play), and lower among those not walking to school.

**Methods:**

Children participating in the UK-wide Millennium Cohort Study wore an Actigraph GT1M accelerometer for seven consecutive days during waking hours. We modelled 6,497 daily PA profiles from singleton children (3,176 boys; mean age: 7.5 years) by means of splines, and used functional analysis of variance to examine the cross-sectional relation of time and place of measurement, demographic and behavioural characteristics to smoothed PA profiles.

**Results:**

Diurnal and time-specific patterns of activity showed significant variation by sex, ethnicity, UK country and season of measurement; girls were markedly less active than boys during school break times than boys, and children of Indian ethnicity were significantly less active during school hours (9:30–12:00). Social activities such as sport clubs, playing with friends were associated with higher level of PA in afternoon (15:00–17:30) and early evenings (17:30–19:30). Lower PA levels between 8:30–9:30 and 17:30–19:30 were associated with mode of travel to and from school, and number of cars in regular use in the household.

**Conclusion:**

Diminished PA in primary school aged children is temporally patterned and related to modifiable behavioural factors. FDA can be used to inform and evaluate public health policies to promote childhood PA.

## Introduction

In the UK, it is recommended that young people participate in activity of moderate to vigorous intensity for at least one hour every day, [1] however only half of seven year old children in the UK achieve these recommended levels, with significant sex, ethnic and geographic variations. [2]

Effective strategies are needed to increase PA levels in young children. However, evidence of their effectiveness is limited. [3] The ecological health approach supports the assumption that more attention should be paid to different correlates of behaviour (e.g., biological, psychological, environmental) during the development of interventions. [4] In consonance with this, recent reviews [5–9] have considered ecological or multilevel perspectives to summarise correlates of physical (in) activity and to inform interventions. The literature on correlates and determinants of physical activity in youth has been judged inconsistent in terms of findings and methodological quality. [8, 10] Notably, a recent review specific to behavioural determinants [6] considers the evidence “limited and inconclusive” for the majority of the determinants. Among the methodological advances advocated [10] there is the need to clarify the “behavioural context”, that is, to highlight key temporal aspects of behavioural characteristics.

Accelerometer-based measures of physical activity (PA) are increasingly used to measure childhood activity levels and their intensity, and to investigate their determinants, in large-scale epidemiological studies. [2, 11, 12] They provide objective estimates of the intensity of movement by measuring accelerations, in one or more directions, of the body segment to which they are attached. In population studies, children are usually asked to wear an accelerometer for several consecutive days during all waking hours, resulting in a large number of measurements for each child. Subsequently, data are typically summarized using total counts, average daily counts per minute and average daily time spent being sedentary or undertaking light, moderate or vigorous activities. [13, 14] However, these summary statistics fail to incorporate important aspects of the structure of the data, [13–15] and do not fully exploit the potential of accelerometry to understand patterns of children’s activity over the course of the day, week and year and their determinants.

Functional data analysis (FDA) is a statistical framework which regards measurements, either discrete or continuous, as generated from continuous, smooth functions. [16] FDA is often used to analyse high-frequency measurements in the temporal domain. [17] The application of FDA as a means to characterise profiles (i.e., patterns or trajectories) of PA and their associations with covariates of interest is a novel approach that has improved our understanding of important predictors of active behaviour. [18, 19]

In this study we applied FDA to model daily profiles of PA in a large sample of seven year old children. Subsequently, we used functional analysis of variance (FANOVA) to examine time and place of measurement, demographic and behavioural characteristics that may explain the variability of daily PA profiles. Our overall objective was to understand temporal patterns of PA according to characteristics of the child, their family and wider environment in order to inform public health interventions designed to increase activity levels in primary school aged children. Specifically we hypothesised that PA levels would be lower in girls than boys at play times and after school, higher in children participating in social forms of exercise (such as sport or play), and lower among those not walking to school.

## Materials and methods

The Millennium Cohort Study (MCS) is a prospective study of the social, economic and health-related circumstances of children living in the UK between September 2000 and January 2002. [20] The original cohort comprised 18 818 children whose parents were first interviewed at home when their children were aged nine months. Five successive contacts were made when children were aged three, five, seven, 11 and 14 years. Children participating in the MCS were invited to wear an accelerometer (Actigraph GT1M [Actigraph, Pensacola, Florida]) at the fourth contact which took place between May 2008 and August 2009 when they were around seven years of age. [2] The MCS contact at age seven and the associated accelerometry study were approved by the Northern and Yorkshire Research Ethics Committee (07/MRE03/32). The analyses reported here did not require additional ethics approval.

Consent to take part in the accelerometry study was given by parents of 12,625 of 14,043 children (90%). Children were asked to wear an accelerometer on their right hip for seven consecutive days during waking hours, except when swimming or bathing as these accelerometers were not waterproof. Data were processed to remove extreme counts, errors due to device malfunctioning or setting, and to perform other quality control checks [21–25]. In this analysis we considered data only from singleton children who wore the accelerometer for at least ten hours a day from 7:00 to 22:00. As a result, we obtained 36,279 daily physical activity profiles from 6,497 singletons (median 6, minimum 1, maximum 18 days per child). To remove within-child correlation and thus simplify analysis, we randomly sampled one daily profile from each child. Finally, we standardised observations to a common time window (8:30–19:30) and then summarised accelerometer measurements in one-minute epochs by aggregating counts originally stored in 15-second epochs.

### Covariates

We considered a number of categorical covariates (Table 1) as potential predictors of the functional PA daily profile. These were classified into three groups: time and place of measurement (country of residence, season of the year, day of the week); socio-demographic (child sex and ethnicity, and maternal socioeconomic and lone parenthood status); and behavioural (children’s screen time, participation in sport/exercise at clubs or school, reading habits, time spent with friends, mode of transport to/from school, and household number of cars/vans in regular use).

**Table 1:** Variables considered in the analysis of determinants of functional day profile.

Variable†	Levels for analysis
***Time and place of measurement factors***	
Country of residence	England; Wales; Scotland; Northern Ireland
Season of measurement*	Winter; Spring; Summer; Autumn
Weekend day of measurement	No; Yes
***Socio-demographic factors***	
Child’s sex	Male or female
Child’s ethnic group	White; Mixed; Indian/Pakistani/Bangladeshi; Black; Other
Maternal socioeconomic status	Higher managerial, administrative and professional occupations; Intermediate occupations; Routine and manual occupations; Never worked and long-term unemployed
Maternal lone parenthood status	Non-lone parent or lone parent
***Behavioural factors***	
Time spent viewing television (hours daily)	≤ 1 hour; > 1 & < 3 hours; ≥ 3 hours
Time spent in sports/activities (club or classes)	Not at all or less than twice a week; 2 days a week; ≥ 3 days a week
Time spent reading for enjoyment	Every day or almost every day; Several times a week or less
Time spent with friends after school	Less than several times a week; Several times a week or more
Mode of transport to/from school	Only car"; "Mixed"; "Only walk/cycling"
Number of cars/vans in regular use per household	0, 1, 2, 3 or more

† Collected at the fourth survey of MCS (age 7 years) with exception of sex and ethnicity, which was captured at the firsy survey.

* Seasons of the year were defined astronomically: Spring (21^st^ March –20^th^ June), Summer (21st June– 20^th^ September), Autumn (21^st^ September– 20^th^ December), and Winter (21^st^ December– 20^th^ March).

With the exception of sex and ethnicity, which were reported at the first MCS survey, all other variables were collected during home interviews at the fourth survey (see [26] for further details).

### Statistical methods

The term FDA comprises statistical methods aimed to model data sampled at high temporal resolution (high-frequency data) in which the individual unit of analysis is the entire continuous function whence the observations have originated. [16, 17, 27] The main characteristic of FDA is that the vector of high resolution data is modelled as a unique functional object typically defined in terms of a spline basis. This functional object provides a mathematical framework which enables exploratory data analysis and inference to be performed using statistical techniques analogous to standard multivariable methods (e.g. ANOVA, regression models, principal component and cluster analysis). [16, 17, 27]

In the description that follows, we outline the notation and basic idea of FDA applied to accelerometer data.

Let *y*_*il*_ denote counts measured by the accelerometer, where *i* = 1,…,*n* is the child for epoch (time) *l* = 1,…,*n*_*i*_. Since in our analysis the time unit was the minute and the time interval was 08:30 to 19:30, there were 660 observations of accelerometer counts available for each child, that is, *n*_*i*_ = 660. Let *y*(*t*) denote a continuous, latent function assumed to be smooth over time, indexed by *t*. FDA assumes that the observed accelerometer counts *y*_*it*_ at a particular time *t* represent a sample from *y*(*t*), possibly measured with error. We used these observations to estimate, by means of splines the latent functions *y*_*i*_(*t*), for each child *i* = 1,…,6497.

The first step is to model observed accelerometer data by means of latent smooth functions *y*_*i*_(*t*) assumed to be smooth over time *t*. The linear predictor

$$y_{i}\left(t\right)=\phi^{\prime }\left(t\right)\;c_{i},$$ (1)

where, **c**_*i*_ is a *k* × 1 vector of coefficients, and *ϕ*(*t*) is a *k*–dimensional basis function system (16) as our starting point. Usually *k* is relatively large thus reflecting the infinite dimensionality of the space of smooth functions where inference takes place in FDA. Within this framework, there are several basis functions *ϕ*_*k*_(*t*) among which one can choose, e.g. Fourier, exponential, truncated power functions, orthogonal polynomials and splines. Here we considered spline functions given their computational efficiency. Among these we used a fourth order cubic *B*–spline basis function system. Numerically, *B*–splines are attractive because they require an amount of computation that increases linearly with the number of observations. [17] Another desirable property of cubic splines is that they are the smoothest possible interpolant through any set of data. [28] This property implies that estimated cubic splines yield the interpolant function that minimize the curvature (i.e. the integral for the second derivative) of the objective function.

Let *ϕ*_*k*_(*t*_*m*_) be the value of the basis function *ϕ*_*k*_ at time *t*_*m*_. The coefficients **c**_*i*_ can be estimated within the generalised linear models (GLM) framework, so that we define:

$$h\left(\mu_{im}\right)=\sum_{k=1}^{K}c_{i,k}\;\phi_{k}\left(t_{m}\right),$$ (2)

where *μ*_*im*_ is the conditional mean of *y*_*im*_, and *h* is a link function. The distribution of the response variable, and the link function are chosen within the generalized linear models (GLM) family. [29]

To account for over-dispersion, which is often found in accelerometry measures, we considered quasi-likelihood models with identity link functions [29]; and linear variance functions of the mean. Lastly, the degree of smoothing, which is given by the dimension *k* of the basis function system, was determined. This was selected using the generalised cross validation (GCV) score which is typically used in nonparametric regression settings:

$$GCV=\left(\frac{n}{n-k}\right)\frac{\sum_{im}{Var({\hat{\mu }}_{im})}^{-1}{\left(y_{im}-{\hat{\mu }}_{im}\right)}^{2}}{n-k}.$$

The number of functions that defined the basis system, *k =* 199, was determined by empirically minimising this score.

We then considered the latent functions *y*_*i*_(*t*) as the outcome in the functional ANOVA model

$$y_{i,g}\left(t\right)=\alpha_{0}\left(t\right)+\alpha_{g}\left(t\right)+\varepsilon_{i,g}\left(t\right),$$ (3)

where the index *g* = 1,…,*G*−1 denotes the level of a categorical covariate different from the baseline category *g* = 0. as described in Ramsay. [16] These parameters have a similar interpretation as in standard ANOVA, however, in functional ANOVA, they are functions of time. The function *α*_0_(*t*) is the accelerometer profile function in the reference category, while the second term, *α*_*g*_(*t*), represents a time-specific contrast of category *g* to the reference category. The error *ε*_*i*,*g*_(*t*) collects unexplained variation, assumed to be independent of the covariates. This FANOVA model [3] was extended to multiple predictors, as described in Ramsay. [16]

Finally, it is interesting to note that a functional parameter can be averaged over a period of time to obtain a scalar ${\bar{\alpha }}_{g}=\int \alpha_{g}(t)\;dt$. Averaged parameters over specific time windows, namely 8:30–9:30; 9:30–12:00; 12:00–13:30; or 17:30–19:30 appear in S1 Table.

We initially performed a univariable FANOVA for each of the considered predictors, using a permutation functional *F* test [17] to assess their association with daily PA profiles. We subsequently included all predictors in the multivariable FANOVA model, which results are presented in this paper. We calculated 95% Confidence Intervals using a bootstrap procedure. Inverse probability weights were used to take into account MCS sampling probability and non-response propensity to the PA study. [30]

### Software

We carried out the analyses in the R programming environment for statistical computing version 3.3.2 (19) [31] in a UNIX platform, using functions from the R package fda. [32]

## Results

**Fig 1:**
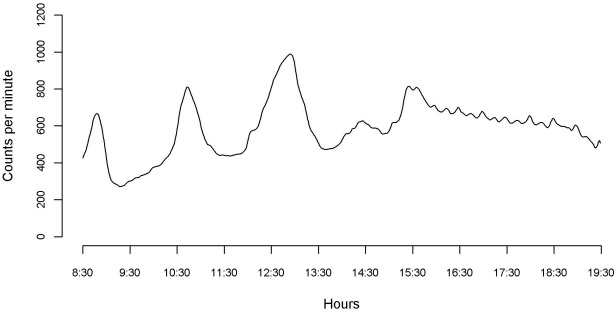
Smoothed mean physical activity function for 6,497 children.

The smoothed mean physical activity function for all children is shown in Fig 1; there are clear periods of increased PA coinciding with journey times to and from school, and lunch and break times.

Functional parameters averaged over the observational period 8:30–19:30 are presented in Table 2; the average of the same parameters over specific time windows, namely 8:30–9:30; 9:30–12:00; 12:00–13:30; or 17:30–19:30, can be found in S1 Table.

**Table 2:** Estimates of total activity by measurement, demographic and behavioural characteristics using time-averaged estimates from multivariable functional analysis of variance (FANOVA).

		Multivariable FANOVA
	*n*	Coefficient (95% CI)
**Country of residence**		
England	4175	Ref
Wales	923	-4.8 (-19.0; 11.1)
Scotland	766	3.3 (-13.2; 20.9)
Northern Ireland	633	-23.0 (-48.6; -2.2)
**Season of measurement**		
Winter	875	Ref
Spring	656	114.3 (93.1; 144.5)
Summer	2777	73.1 (54.7; 90.9)
Autumn	2189	36.7 (17.4; 55.9)
**Weekend day of measurement**		
No	5065	Ref
Yes	1432	5.7 (-7.9; 19.5)
**Child’s sex**		
Male	3176	Ref
Female	3321	-69.9 (-79.7; -58.2)
**Child’s ethnic group**		
White	5711	Ref
Mixed	168	-36.6 (-67.8; -2.8)
Black	142	14.6 (-20.8; 62.9)
Indian/Pakistani/Bangladeshi	386	-38.9 (-63.9; -19.3)
Other	90	-64.2 (-115.3; 5.6)
**Maternal socioeconomic status**		
Never worked and long-term unemployed	268	Ref
Routine and manual occupations	2582	3.5 (-14.0; 17.8)
Intermediate occupations	1201	3.1 (-20.8; 23.1)
Higher managerial, administrative and professional occupations	2446	6.0 (-16.8; 34.1)
**Maternal lone parenthood status**		
Non-lone parent	5485	Ref
Lone parent	989	8.5 (-17.0; 37)
**Time spent viewing television (hours/day)**		
≤ 1	1337	Ref
> 1 & < 3	4205	8.4 (-5.0; 20.6)
≥ 3	948	12.4 (-4.3; 33.4)
**Time spent in sports/activities (club or classes)**		
Not at all or less than twice a week	3370	Ref
2 days a week	1576	6.0 (-6.5; 20.6)
3 or more days a week	1544	30.5 (18.4; 44.4)
**Time spent reading for enjoyment**		
Several times a week or less	3806	Ref
Every day or almost every day	2682	-15.0 (-27.1; -6.6)
**Time spent with friends after school**		
Less than several times a week	3441	Ref
Several times a week or more	2674	26.0 (13.5; 36.8)
**Mode of transport to/from school**		
Only walking/cycling	3030	Ref
Mixed	376	5.0 (-13.6; 28.0)
Only car	3074	-15.4 (-27.4; -1.3)
**Number of cars/vans in regular use per household**		
0	579	Ref
1	2304	-22.2 (-46.0; -2.0)
2	3230	-39.5 (-64.6; -18.0)
3+	374	-54.6 (-85.6; -21.9)

**Fig 2:**
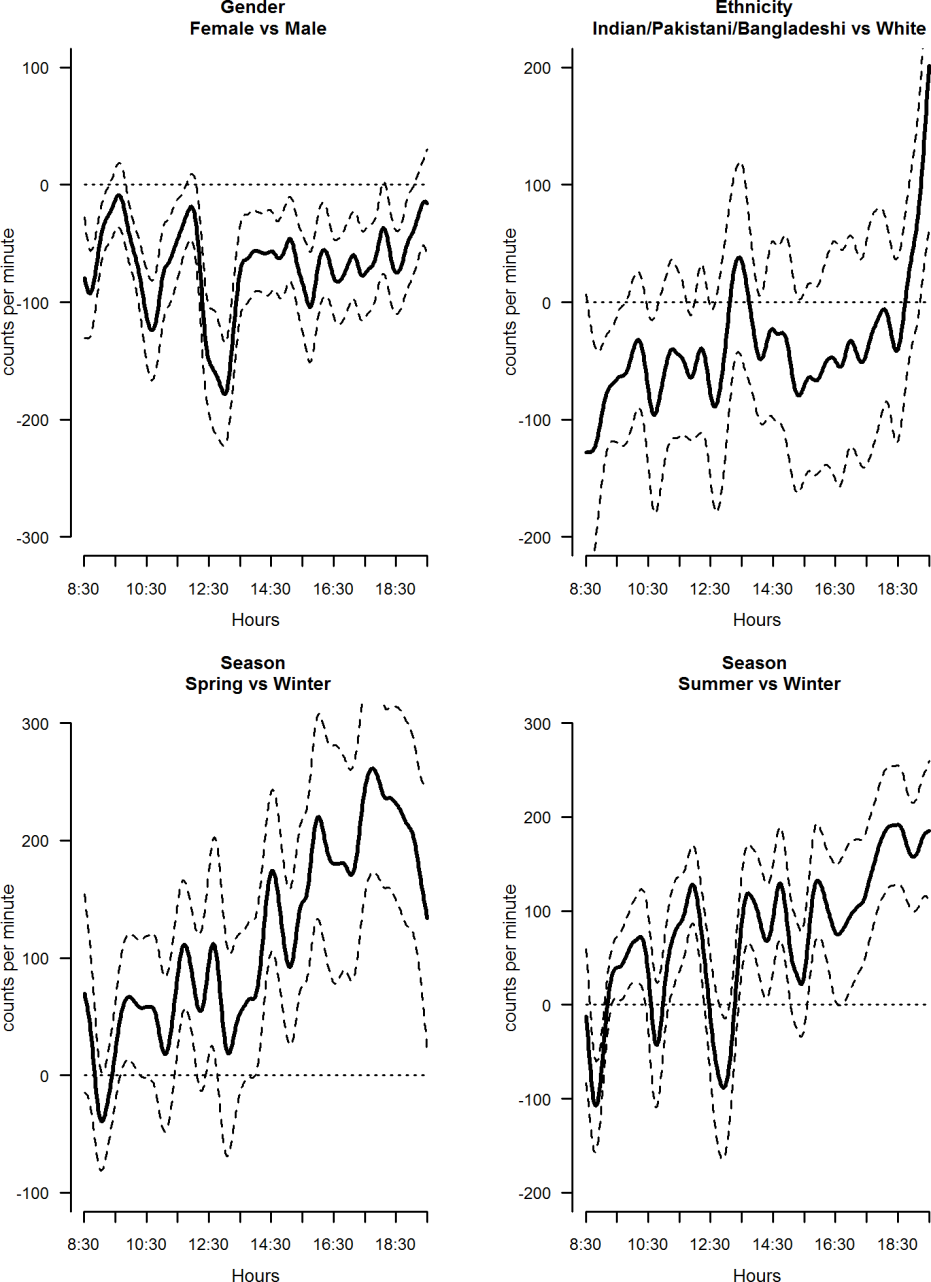
Estimated coefficient functions, with 95% bootstrap confidence intervals, for sex, ethnicity and season.

**Fig 3:**
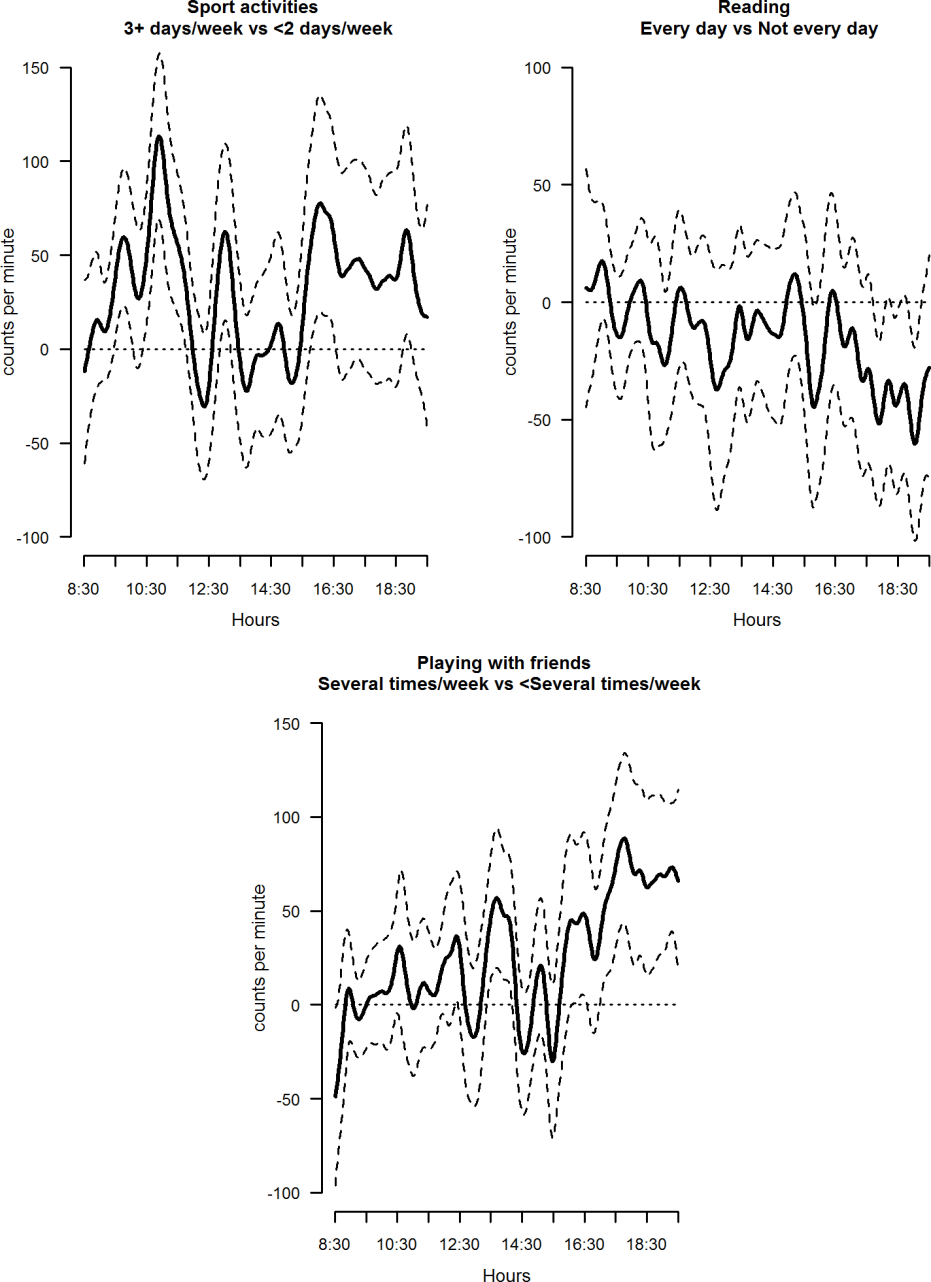
Estimated coefficient functions, with 95% bootstrap confidence intervals, for sport, reading and friends.

**Fig 4:**
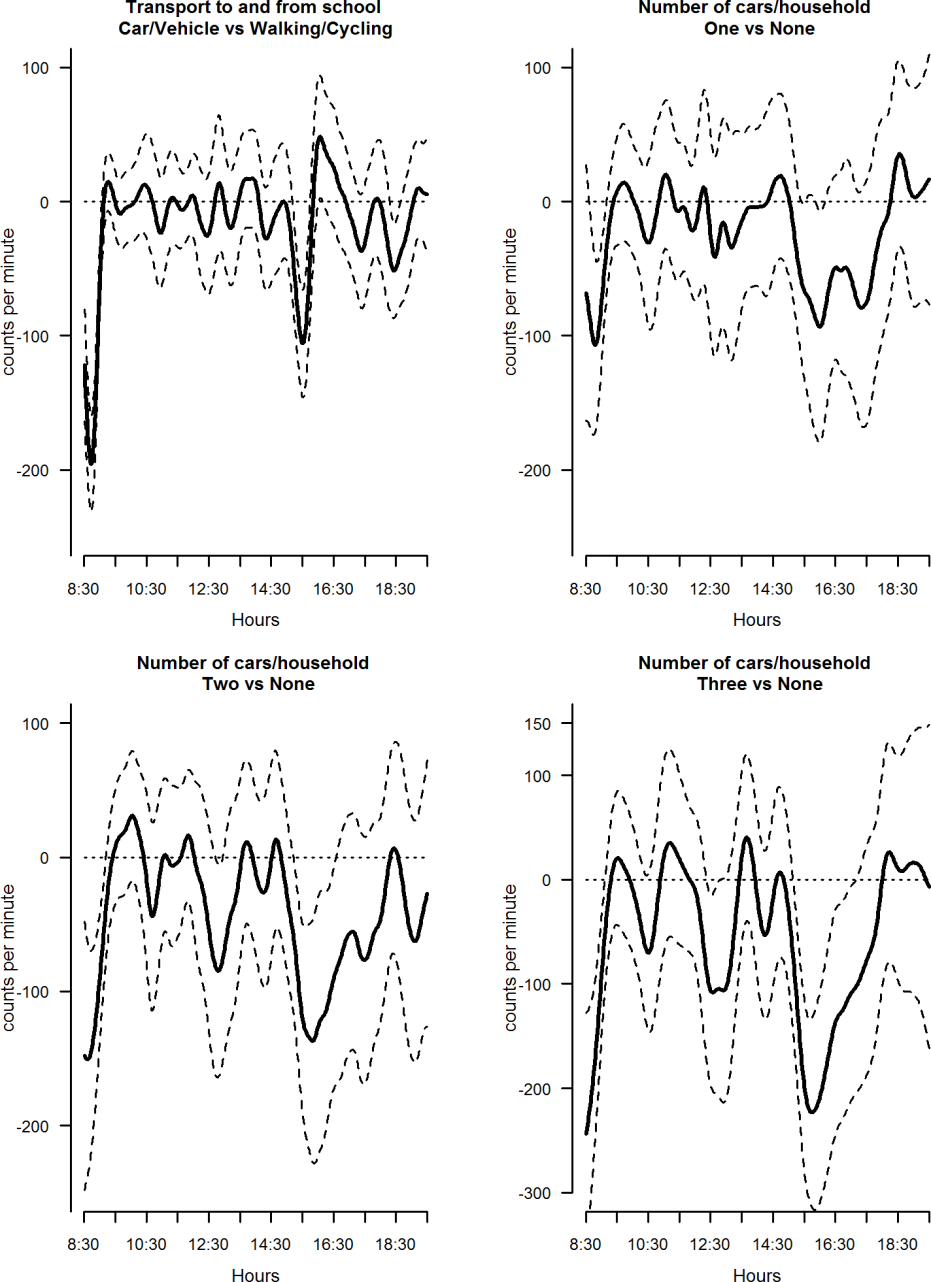
Estimated coefficient functions, with 95% bootstrap confidence intervals, for mode of transport to and from school and number of cars/vans of regular use in the household.

The mutually adjusted daily PA profiles of all the explanatory variables considered varied significantly across categories: examples of these are shown in Figs 2–4.

Children living in Northern Ireland were significantly less active than those living in England. Activity levels in girls were consistently lower than those of boys throughout the day, with the greatest differences apparent between 12:00 and 13:30 (Fig 2) where the averaged functional parameter is -128.5 95%CI (-145.5; -107.3) (S1 Table). A difference was also observed by ethnic group, with children from an South Asian (Fig 2) or mixed ethnic groups (Table 2), being significantly less active during school hours but more active before bedtime relative to children from a white ethnic group. Children were more active across the day in spring and summer (Fig 2) than winter, with the greatest differences observed in the afternoon/evening hours (S1 Table). Differences in total physical activity levels were observed between the weekend and weekdays, with high levels of activity observed in weekend days between 9:30 and 12:00 and during the early afternoon (between 13:30 and 15:00) (S1 Table). Interestingly, these higher levels of activity were compensated for by lower levels of activity during the early morning and lunch time period.

Children who participated in organised sports and activities (club or classes) three or more times a week showed generally higher activity levels across the day, especially during periods that might coincide with school play times (Fig 3); in contrast, children who read for enjoyment every day or almost every day tended to have lower activity levels confined to the late afternoon / early evening compared to those who read less frequently or not at all (Fig 3). Children whose parents reported that they played with friends several times a week or more after school were more active during the early evening compared to those who did not or did so less frequently (Fig 3), with an average functional parameter equal to 73.0 95%CI (46.2; 96.5) (S1 Table).

Mode of transport to and from school (Fig 4), and number of vehicles in regular use in the household, were also significantly associated with daily PA profiles (Fig 4). Children who never walked or cycled to and from school were significantly less active than those who did some or all of the time at the start and end of the school day, as were those living in households with one or more cars or vans in regular use. Notably, children that used cars or other vehicles to go to school have an average functional parameter equal to -75.9 95%CI (-93.2; -60.1) in the time window 8:30–9:30 (S1 Table). Similarly, children living in households with three cars shows a functional parameter equal to -98.8 95%CI (-142.8; -54.0) in the same time window (S1 Table).

## Discussion

### Summary of findings

This study is, to our knowledge, the largest study to use FDA and FANOVA for the analyses of accelerometer-based measures of physical activity. Our findings provide additional insights into variations in children’s activity levels across their waking day. They confirm and extend previous observations that girls are less active and identify periods in the school day where these differences are most apparent. Children whose measurements were obtained in the spring and summer months were significantly more active than those measured in winter, after adjusting for other factors. The importance of social activities for physical activity is underscored by the time-specific differences in activity levels observed in those involved in sport or after school clubs or who played with friends on a regular basis. While children who read for enjoyment were less active than those who did not, from a developmental perspective reading is clearly a beneficial activity and these findings do not suggest a detrimental influence of reading *per se*. Our findings also extend the existing strong evidence that cycling or walking to school on some or all days of the week is an effective strategy to get children more active and therefore supports schemes encouraging this, such as the UK ‘Walk to School’ campaign. We have demonstrated the value of using FDA to condense the extensive information available from accelerometer-derived measures of total physical activity in a population-based cohort by proposing a novel method to determine the spline basis functions, and using quasi-likelihood procedures to estimate the models’ functional parameters.

### Comparisons with other findings

Among the methodological advances advocated [10, 33] there is the need to increase the context specificity on which the correlates are defined. Clarification of the behavioural context could enhance the specificity of behavioural models, with key characteristics of context being person, place, time and activity. Related to this research agenda, there has also been increased interest in the temporal dimension of correlates of physical activity. These studies have been reviewed [34, 35]; Stanley and colleagues [35] considered studies conducted on children (8–14 years) between 1990 and 2011. They focused on studies that evaluated determinants at school break time and after school. The authors found some correlates with consistent associations with physical activity; in particular, sex (with boys more active) was associated with physical activity in both periods. Brooke and colleagues [34] reviewed differences in physical activity in school aged children across different time periods (weekdays vs weekends, in school vs out of school, weekends vs out of school and lesson time vs break time). They found that children were more active on weekdays than during the weekend, while the unit of physical activity measurement was found to influence comparisons across other time-periods. We have found a small number of other papers also exhuming the temporal characteristics of physical activity correlates and published after this review [36–40]. Hesketh and colleagues [36] have previously examined patterns of activity across the day in a sample of 593 preschool-aged children living in Southampton, UK. Their time-specific observations demonstrated the importance of considering temporality for physical activity interventions targeting, for example, sex differences in the morning when girls were less active than boys. Similarly, a study by Li and colleagues [38] in 1075 8-11-year-old children has shown daily variation in activity levels, with periods of higher activity during the commute to school times, morning break and lunch-times. Stanley and colleagues [39] analysed accelerometer data from 423 south Australian children aged between 10 and 14 years. The authors used the correlation component regression models to predict total physical activity during lunch-time and after-school periods for boys and girls separately; they were able to explain 25% and 17% of the variance for boys and girls respectively during lunch time, while the after-school model predicted 20% and 7% of the variability in boys and girls, respectively. Hubbard and colleagues [37] evaluated the difference in different physical activity dimensions between boys and girls, and normal and overweight/obese children in 517 US school children with a mean age of 9 years. They found lower levels of physical activity in girls, and overweight/obese children, in all of the periods investigated (school, weekday out-of-school, weekend). Finally, De Baere and colleagues [40] examined temporal patterns of both physical activity and sedentary behaviour in 211 children aged 10–14 and reported on particularly active periods of the day, like those reported in our study, but also more sedentary periods and periods where these types of activity compete.

Regarding, statistical methods, in another study based on a small sample of school children in the United States, Fan and colleagues [18] used FDA to assess PA differences between adolescent girls in different school years and with high versus low body mass index (BMI) categories. The sample comprised fewer than one hundred pupils in each of the three year groups examined. The authors reported lower activity levels in grade 10 (15 to 16 years) than grade 11 (16 to 17 years) and 12 (17 to 18 years) girls during evening hours; differences in activity levels were also observed for grade 10 girls with low- versus high-BMIs during the morning period, with the low BMI group being more active. Our study did not re-examine the impact of BMI status, but we do concur with Fan and colleagues [18] on the importance of FDA in enhancing this field of research. Another analytic approach has been outlined by Morris and colleagues [41] who used a wavelet-based functional model incorporating covariates and random effects to analyse 550 accelerometer profiles from 112 early adolescents and demonstrated irregular profiles characterised by many peaks representing short bursts of intense activity. The main focus of the study by Morris and colleagues was to sample stochastically missing regions of incomplete profiles, which were not present in our dataset.

In summary we have found the evidence inconclusive, apart from consistently higher levels of physical activity observed in boys across all time periods examined. While patterns of physical activity within and between days have been studied, few studies have analysed correlates associated with different levels of activity within a single day.

### Strengths and limitations

There is increasing interest in using the internal clock of the accelerometer to depict daily PA patterns. [42–44] The representation of accelerometer data as a functional data object over time gives a clearer picture of the daily PA patterns. This enables researchers to identify contextual information for periods when children are most or least active. These daily patterns can be identified at a population level using functional descriptive statistics, such as the functional mean.

Linkage of the activity data to a wide range of individual and behavioural information from MCS enabled examination of the effects of these covariates and activity levels and temporal patterns. A strength of our study is the novel application of FANOVA to explain the variability of fitted functional daily PA patterns, in this study using a sample of approximately 6500 daily physical activity profiles. This has allowed us to extend, in a large population-based study of primary school-aged children, our earlier observations of reduced physical activity among girls, those who do not engage in sporting or other social activities, and those who do not ever walk or cycle to school by demonstrating the time of day where these differences are most marked. We believe this provides additional evidence as well as a methodological approach with which to inform and evaluate population level strategies to increase children’s physical activity.

A potential limitation of using accelerometers to define activity is that it underestimates activities that do not include vertical movement of the trunk, such as cycling, and, for the device used in this study, aquatic activities such as swimming. We also did not use all information available on each child; this choice was made to ensure independence of daily profiles, since the methods developed by Ramsay and Silverman [17] as implemented in the R package fda require independence of functional profiles. Further work is needed to develop and evaluate efficient FDA methods that consider dependence structures on the functional profiles in large scale population studies.

FDA requires a common observation time without missing data. The observation period between 8:30 and 19:30 was chosen to maximise the sample size without missing data in the observation period. By using this time window, we may have missed some active transportation to school; however, the average of the functional coefficient between the period 8:30 and 9:30 shows a lower level of physical activity and it seems that some of the effect of active travelling during the before school time period was captured by the functional coefficient (See Fig 4).

Our analysis did not consider interactions between biological and behavioural determinants, this is a limitation of our work as it is important to understand and evaluate how different levels (and types) of determinants interact to promote healthy behaviours; doing this within FDA requires complex computational considerations but this should be considered in future work.

### Implications for research and practice

The description and the analysis over time of factors that affect PA patterns can provide information useful for planning tailored public health interventions aimed at increasing PA among children. For example, several studies have highlighted the lower levels of activity in primary school-aged girls, [5] however quantifying these differences more precisely over the waking day, particularly during school time, can be used to inform and evaluate school-based interventions designed to increase participation in more intense PA. For example, our findings suggest that initiatives aimed at increasing activity during the lunchtime period may be particularly beneficial for girls.

Our observation of higher levels of PA in summer and spring, has been previously reported. [45, 46] This supports the implementation of physical activity promotion interventions in children during winter, possibly during the afternoon when the greatest differences are observed relative to activity levels in summer. The approach used in this study enables research to identify and evaluate mediators of seasonal variation in activity, such as social, organisational, and environmental factors, to inform the design of interventions to reduce this variation. [45]

Our findings about the temporal effects on PA of mode of transport to school underscore the importance of policy initiatives to support active walking to school schemes. [47]

Participation in sporting and other social activities outside of school is associated with higher activity levels throughout the day. While the causal direction of these associations cannot be established in an observational study, public health measures are urgently needed to overcome barriers to involvement and to encourage participation in sports and other activities (such as for example dancing) in and out of school, as proposed in the government’s recently published obesity strategy. [48] Finally, we have shown that children who play with friends more often are more active–social aspects of activity and the influence of peer networks should be considered when tailoring interventions to increase activity in children.

### Conclusions

We have presented methods based on FDA applied to a large population-based study of children with individual PA profiles based on accelerometer data. These methods are useful to model the temporal and population heterogeneity of physical activities. In particular, our methodological approach and findings related to modifiable behavioural factors may be used to inform and evaluate population-based strategies aimed at increasing activity levels.

## Supporting information

S1 Table Estimates of total activity by measurement, demographic and behavioural characteristics functional parameters (FANOVA) averaged over specific day time windows; 8:30–9:30; 9:30–12:00; 12:00–13:30; or 17:30–19:30. (DOCX)
