# Supplementary material for: Using functional data analysis to understand daily activity levels and patterns in primary school-aged children: Cross-sectional analysis of a UK-wide study
Source: PLoS One. 2017 Nov 8;12(11):e0187677. doi: 10.1371/journal.pone.0187677 (PMC5678875; doi:10.1371/journal.pone.0187677)
Supplement: S1 Table — (DOCX) [file pone.0187677.s001.docx]

**Table S1.** Estimates of total activity by measurement, demographic and behavioural characteristics functional parameters (FANOVA) averaged over specific day time windows; 8:30-9:30; 9:30-12:00; 12:00-13:30; or 17:30-19:30.

|  |  | 8:30-9:30 | 9:30-12:00 | 12:00-13:30 | 13:30-15:00 | 15:00-17:30 | 17:30-19:30 |
| --- | --- | --- | --- | --- | --- | --- | --- |
|  | **n** | **Coef**  **95%CI** | **Coef**  **95%CI** | **Coef**  **95%CI** | **Coef**  **95%CI** | **Coef**  **95%CI** | **Coef**  **95%CI** |
| **Child’s sex** |  |  |  |  |  |  |  |
| Male | 3176 | Ref | Ref | Ref | Ref | Ref | Ref |
| Female | 3321 | **-54.1**  **(-72.8; -36.7)** | **-60.8**  **(-73.2; -44.4)** | **-128.5**  **(-145.5; -107.3)** | **-60.7**  **(-77.6; -41.3)** | **-74.1**  **(-90.2; -50.6)** | **-51.6**  **(-70.2; -31.9)** |
| **Weekend day of measurement** |  |  |  |  |  |  |  |
| No | 5065 | Ref | Ref | Ref | Ref | Ref | Ref |
| Yes | 1432 | **-213.9**  **(-232.1; -198.7)** | **100.4**  **(81.5; 127.1)** | **-193.4**  **(-216.4; -173.2)** | **137.0**  **(113.7; 164.7)** | 26.3  (-2; 47.3) | 22.1  (-1.7; 44.7) |
| **Season** |  |  |  |  |  |  |  |
| Winter | 875 | Ref | Ref | Ref | Ref | Ref | Ref |
| Spring | 656 | 2.3  (-28; 27) | **61.8**  **(31.3; 88.5)** | **60.8**  **(20.6; 107.8)** | **111.7**  **(68.7; 146.3)** | **171.0**  **(124.4; 222.9)** | **221.1**  **(171.2; 264.7)** |
| Summer | 2777 | **-36.6**  **(-58.5; -8.6)** | **54.2**  **(28.3; 77.3)** | -1.6  (-40.4; 32.4) | **101.5**  **(62.3; 133.1)** | **86.8**  **(49.7; 122.7)** | **169.9**  **(142.2; 208.4)** |
| Autumn | 2189 | **36.1**  **(10.7; 66.8)** | 20.3  (-4; 51.6) | **50.4**  **(11.2; 92)** | 24.5  (-57.5; 8.8) | **55.7**  **(19.7; 92.2)** | **70.7**  **(34.6; 102.3)** |
| **Country** |  |  |  |  |  |  |  |
| England | 4175 | Ref | Ref | Ref | Ref | Ref | Ref |
| Wales | 923 | -19.3  (-43.7; 3.6) | -12.6  (-29.4; 6.8) | -29.8  (-57.9; 1.6) | **34.8**  **(10; 63.1)** | -21.2  (-45.1; 5.3) | 19.4  (-4.4; 48.7) |
| Scotland | 766 | -15.2  (-35.4; 4.4) | 22.7  (-7.5; 47) | -9.4  (-41.8; 22) | -15.4  (-48; 14.8) | -5.3  (-38.9; 26.1) | 25.4  (-9.2; 64.1) |
| Northern Ireland | 633 | **-63.6**  **(-85.8; -42.1)** | **-58.4**  **(-80.4; -37.4)** | **-121.3**  **(-160.8; -83.7)** | 3.8  (-48.4; 37.2) | -6.0  (-53.5; 34.7) | **73.3**  **(28.3; 112.8)** |
| **Child’s ethnic group** |  |  |  |  |  |  |  |
| White | 5711 | Ref | Ref | Ref | Ref | Ref | Ref |
| Mixed | 168 | **-93.9**  **(-138.2; -51.8)** | 32.3  (-84.7; 11.1) | -14.5  (-85.2; 56.2) | -33.6  (-92.2; 29.8) | -61.2  (-99.5; 16.4) | 3.2  (-64.4; 61.2) |
| Black | 142 | -1.9  (-44.9; 44.5) | 29.4  (-25.6; 85.4) | 59.9  (-9.1; 146.2) | -8.7  (-70.9; 80.7) | 27.8  (-43.6; 122.2) | -33.6  (-88.5; 34.3) |
| Indian/Pakistani/Bangladeshi | 386 | **-98.3**  **(-147.9; -47)** | **-58.5**  **(-95.5; -20.1)** | -35.1  (-74.9; 7.2) | -22.8  (-57.6; 15) | -57.0  (-101.9; -6.7) | 18.6  (-30; 65.4) |
| Other | 90 | -63.6  (-156.6; 25) | -46.4  (-104.2; 32.7) | -20.9  (-147.3; 140.4) | -66.3  (-139.6; 46.3) | **-90.7**  **(-213; -12.7)** | -67.5  (-144.1; 22.9) |

|  |  | 8:30-9:30 | 9:30-12:00 | 12:00-13:30 | 13:30-15:00 | 15:00-17:30 | 17:30-19:30 |
| --- | --- | --- | --- | --- | --- | --- | --- |
|  | **n** | **Coef**  **95%CI** | **Coef**  **95%CI** | **Coef**  **95%CI** | **Coef**  **95%CI** | **Coef**  **95%CI** | **Coef**  **95%CI** |
| **Maternal socioeconomic status** | | |  |  |  |  |  |
| Never worked and long-term unemployed | 268 | Ref | Ref | Ref | Ref | Ref | Ref |
| Routine and manual occupations | 2582 | **-25.5**  **(-49.9; -8.5)** | -3.0  (-24.8; 14.8) | -3.6  (-35.8; 22.6) | -1.9  (-29.8; 23.6) | 8.2  (-22.4; 32.6) | **31.1**  **(0.9; 61.4)** |
| Intermediate occupations | 1201 | 39.3  (-10.8; 96.7) | 26.5  (-9.6; 61.4) | 9.2  (-41.8; 60) | 10.8  (-35.9; 42.4) | -14.8  (-69; 39.5) | -31.8  (-91.5; 33.7) |
| Higher managerial, administrative and professional occupations | 2446 | **72.5**  **(16.3; 132.4)** | 26.8  (-4.9; 62.2) | 44.6  (-10.8; 96.3) | 1.5  (-50.4; 45.4) | -22.8  (-78.3; 35.3) | -32.7  (-98.8; 26.1) |
| **Maternal lone parenthood status** | | |  |  |  |  |  |
| Non-lone parent | 5485 | Ref | Ref | Ref | Ref | Ref | Ref |
| Lone parent | 989 | **89.3**  **(37; 141.6)** | **45.1**  **(1.1; 85.5)** | 29.7  (-26.6; 75.6) | 14.8  (-43.7; 55.1) | -9.2  (-63.9; 44.6) | -71.2  (-138.6; 4.2) |
| **Time spent in sports/activities (club or classes)** | | |  |  |  |  |  |
| Not at all or less than twice a week | 3370 | Ref | Ref | Ref | Ref | Ref | Ref |
| 2 days a week | 1576 | 1.5  (-13.9; 19.6) | **20.0**  **(2.7; 39)** | 2.6  (-21.7; 29.8) | **19.4**  **(1.6; 48.4)** | -1.5  (-25.4; 26.9) | -4.6  (-32; 22.4) |
| 3 or more days a week | 1544 | 11.3  (-6.6; 30.3) | **58.0**  **(39.9; 73.9)** | 15.1  (-11.2; 32.9) | -3.4  (-30.1; 22.2) | **40.5**  **(16.4; 67.8)** | **36.8**  **(5.3; 71.9)** |
| **Time spent reading for enjoyment** | | |  |  |  |  |  |
| Several times a week or less | 3806 | Ref | Ref | Ref | Ref | Ref | Ref |
| Every day or almost every day | 2682 | 6.5  (-10.2; 21.2) | -8.0  (-21.4; 5.6) | -20.5  (-40.6; 1.8) | -9.8  (-31.8; 8.1) | -15.4  (-33.8; 1.7) | **-43.2**  **(-66; -19.2)** |
| **Time spent with friends after school** | |  |  |  |  |  |  |
| Less often than several times a week | 3441 | Ref | Ref | Ref | Ref | Ref | Ref |
| Several times a week or more | 2674 | -10.0  (-27.5; 8) | 10.1  (-2.8; 24.3) | 13.2  (-2.5; 33.2) | **19.7**  **(0.2; 34.2)** | **29.4**  **(9.6; 51.6)** | **73.0**  **(46.2; 96.5)** |

|  |  | 8:30-9:30 | 9:30-12:00 | 12:00-13:30 | 13:30-15:00 | 15:00-17:30 | 17:30-19:30 |
| --- | --- | --- | --- | --- | --- | --- | --- |
|  | **n** | **Coef**  **95%CI** | **Coef**  **95%CI** | **Coef**  **95%CI** | **Coef**  **95%CI** | **Coef**  **95%CI** | **Coef**  **95%CI** |
| **Mode of transport to and from school** | | |  |  |  |  |  |
| Only walking/cycling | 3030 | Ref | Ref | Ref | Ref | Ref | Ref |
| Mixed | 376 | -24.2  (-54.5; 8.3) | 14.1  (-18.2; 46.4) | -28.6  (-61.2; 2.4) | 13.9  (-30; 54.2) | 6.4  (-28.3; 51.5) | 21.5  (-16.6; 63.5) |
| Only car | 3074 | **-75.9**  **(-93.2; -60.1)** | -3.0  (-16.1; 11.6) | -8.9  (-26.7; 13.4) | -2.7  (-23.9; 23.5) | -14.7  (-37; 9.4) | -17.7  (-34.8; 5.5) |
| **Number of cars/vans in regular use per household** | | |  |  |  |  |  |
| 0 | 579 | Ref | Ref | Ref | Ref | Ref | Ref |
| 1 | 2304 | **-57.1**  **(-90; -26.7)** | -4.4  (-32.6; 20.5) | -18.5  (-66.8; 21.6) | 2.8  (-38; 38.6) | **-59.6**  **(-108.1; -18.9)** | -1.0  (-51.5; 42) |
| 2 | 3230 | **-86.2**  **(-108.5; -46.4)** | 0.4  (-23.7; 29.8) | -48.5  (-81.8; 0.5) | -4.7  (-47.7; 34) | **-88.3**  **(-138.9; -45)** | -34.7  (-89.6; 7.5) |
| 3+ | 374 | **-98.8**  **(-142.8; -54)** | -8.4  (-45.5; 40.2) | **-75.6**  **(-132.9; -18.7)** | -4.0  (-56.3; 36.6) | **-142.7**  **(-200; -92.4)** | -3.5  (-75.7; 73.6) |
| **Time spent viewing television (hours daily)** | | |  |  |  |  |  |
| ≤ 1 hour | 1337 | Ref | Ref | Ref | Ref | Ref | Ref |
| > 1 & < 3 hours | 4205 | -5.2  (-28.6; 13.5) | -1.9  (-17.8; 12.5) | 18.1  (-14.5; 42.1) | 7.9  (-9.3; 27.9) | 14.5  (-9.4; 32.7) | 18.8  (-15.6; 42.5) |
| ≥ 3 hours | 948 | -8.1  (-35.5; 17.8) | 6.7  (-13.2; 30.9) | **42.8**  **(8.3; 83.9)** | **39.5**  **(3.5; 70.1)** | 7.0  (-25.5; 26.8) | 6.2  (-40.2; 43.3) |
